# A real-world study of treatment sequences and second-line clinical outcomes in patients with HER2-positive metastatic breast cancer in US community practice

**DOI:** 10.1007/s10147-024-02492-5

**Published:** 2024-03-25

**Authors:** Della Varghese, Giovanna I. Cruz, Colden Johanson, Liz Toland, Miguel Miranda, Eleanor C. Faherty, David Harland, Henry G. Kaplan

**Affiliations:** 1 AstraZeneca Pharmaceuticals, Gaithersburg, MD USA; 2 Syapse Holdings, Inc., 1442 Pottstown Pike, Unit #3008, West Chester, PA 19380 USA; 3 AstraZeneca Pharmaceuticals, Cambridge, UK; 4 Swedish Cancer Institute, Seattle, WA USA

**Keywords:** HER2-directed therapy, Metastatic breast cancer, Real-world, Epidemiology

## Abstract

**Purpose:**

Standard-of-care for HER2-positive metastatic breast cancer (HER2 + mBC) patients consists of trastuzumab ± pertuzumab with chemotherapy in first-line (1L), and ado-trastuzumab emtansine (T-DM1) or the more recently approved trastuzumab deruxtecan (T-DXd) in second-line (2L). Contemporary data on treatment sequencing and real-world effectiveness is limited. This study aims to report 2L treatments and outcomes among HER2 + mBC patients in the United States (US).

**Methods:**

HER2 + mBC patients initiating 2L treatment (index date) between January 2014 and February 2021 were identified from the Syapse Learning Health Network (LHN) database. Summary statistics for patient characteristics, treatment received, reasons for 2L discontinuation and time to 2L-clinical outcomes are reported.

**Results:**

Of the 312 patients initiating 2L treatment, had a median age of 59 years (interquartile range [IQR], 50–66) at the start of 2L. The majority were white (69%) and had de novo mBC (62%). Top three 2L regimens included T-DM1 ± endocrine therapy (29%), trastuzumab/pertuzumab/taxane (10%) and T-DM1/trastuzumab (8%). Around 88% discontinued 2L and 63% received subsequent treatment. Median time-to-next-treatment was 10.6 months (95% CI, 8.8–13.3) and real-world progression-free-survival was 7.9 months (95% CI, 7.0–9.9). Among 274 patients who discontinued 2L, 47% discontinued due to progression and 17% because of intolerance/toxicity, respectively.

**Conclusion:**

This real-world US study showed that approximately two-thirds of 2L patients received subsequent therapy and disease progression was the most common reason for 2L discontinuation highlighting the need for timely 2L treatment with the most efficacious drug to allow patients to achieve longer treatment duration and delayed progression.

**Supplementary Information:**

The online version contains supplementary material available at 10.1007/s10147-024-02492-5.

## Introduction

Human epidermal growth factor receptor 2 (HER2)-overexpressing breast cancer (HER2 + tumors) is an aggressive subtype that has been associated with increased risk of recurrence, metastasis, and mortality [1]. Contemporary data from the United States estimate that approximately 15–20% of breast cancers are HER2 + and 6% of women present with metastatic disease at initial diagnosis [2, 3]. De novo and recurrent disease each account for half of women with HER2 + metastatic breast cancer (HER2 + mBC) [4]. Prior to the introduction of HER2-targeted therapies, median overall survival (OS) of patients diagnosed with HER2 + mBC was 15 months with traditional chemotherapy [5].

The US Food and Drug Administration’s (FDA) 1998 approval of the anti-HER2 monoclonal antibody trastuzumab [6], and subsequent approvals of other HER2-targeted therapies have redefined the treatment landscape for patients with HER2 + breast cancer, as these therapies offered improved progression-free survival (PFS) and overall survival (OS) compared to chemotherapy alone [7–12]. The median survival of patients diagnosed after the introduction of HER2-targeted therapies is about 34 months from metastatic diagnosis [13]. Monoclonal antibodies work best in combination with chemotherapy [14]. The standard-of-care treatment for patients with HER2 + mBC has consisted of trastuzumab ± pertuzumab along with chemotherapy in the first-line (1L), followed by ado-trastuzumab emtansine (T-DM1) in second-line (2L). T-DM1, an antibody drug conjugate (ADC), was approved in 2013 after demonstrating improved median PFS and OS over lapatinib plus capecitabine in EMILIA, a Phase III randomized controlled trial of patients with HER2 + advanced breast cancer previously treated with trastuzumab and a taxane (PFS: 9.6 vs. 6.4 months; OS: 30.9 vs. 25.1 months) [15]. The superior efficacy of T-DM1 compared to physician’s choice was confirmed in heavily pre-treated patients in the TH3RESA trial that showed improved PFS of 6.2 months compared to 3.3 months and OS of 22.7 months versus 15.8 months [16, 17]. Benefits of ADCs are their ability to deliver their cytotoxic payload to cells recognized by the antibody component, thereby minimizing damage to normal cells leading to reduced systemic side effects [14].

More recently in 2022 a second ADC, trastuzumab deruxtecan (T-DXd), received FDA approval in 2L after the DESTINY-Breast 03 trial demonstrated a significant reduction in the risk of disease progression compared with T-DM1 in 2L [18]. The second interim analysis confirmed the benefit of T-DXd over T-DM1; median PFS 28.8 months vs 6.8 months and although median survival has not been reached in either arm, the hazard ratio for overall survival favors T-DXd (HR 0.64; 95% CI 0.47–0.87) [18, 19]. Both the National Comprehensive Cancer Network (NCCN) and the European Society of Medical Oncology (ESMO) treatment guidelines list T-DXd as the preferred 2L therapy for HER2 + mBC and T-DM1 as a recommended therapy [20–22]. Contemporary real-world data on treatment sequencing and clinical outcomes beyond first-line is limited [23–27] and is of particular interest in light of the changes in the treatment landscape for 2L HER2 + mBC. The goal of this retrospective real-world study was to understand the current treatment landscape among HER2 + mBC patients following the 1L treatment and associated 2L clinical outcomes. This data is critical in understanding if eligible patients receive optimal and timely targeted therapies.

## Methods

### Study design and database

This retrospective cohort study includes patients from the Syapse Learning Health Network (LHN), a longitudinal database of patients receiving cancer care in US community-based integrated-care delivery networks. The LHN consists of approximately 460 hospitals, 1350 oncologists across 25 states with over 216,000 newly diagnosed patients per year. Each patient’s record includes data from multiple sources: electronic health records (EHR), laboratory and radiology/imaging systems, hospital-based cancer registries, computerized order entry systems, commercial laboratories, and other external sources to capture the patient journey from pre-diagnosis to death; additional data is captured from her EHR by Certified Tumor Registrars (CTRs).

### Study population

Study population consisted of HER2-positive (HER2 +) mBC patients with CTR-enriched data, who were 18 years or older at time of metastatic diagnosis and received 2 or more lines of therapy (LoT) in the metastatic setting. Eligible patients initiated second-line therapy (2L) between January 2014 and February 2021 (index date) and had evidence of two or more clinical encounters in the EHR with at least one after the index date. HER2 status was determined by a documented positive immunohistochemistry (IHC) or fluorescence hybridization in situ (FISH) test within 60 days before or 45 days after the mBC diagnosis. Patients with an unknown HER2 test date were included if the patient was HER2-positive at mBC diagnosis per hospital registry data. The study objectives were also addressed in a sub-cohort of patients who received a trastuzumab-based 2L regimen (T-based 2L) due to its frequent use as a therapy beyond T-DM1 in order to investigate similarities and differences to other 2L-treated patients.

### Baseline patient and treatment characteristics

Baseline characteristics at initial diagnosis included race, ethnicity, sex, region of residence, menopausal status, stage and histology. Metastatic presentation was categorized as de novo (patient had stage IV disease at initial diagnosis) or recurrent. Clinical characteristics at index date included hormone receptor status, sites of distant metastasis, and age. Tumor hormone-receptor positive status was determined based on a positive estrogen-receptor (ER) and/or progesterone-receptor (PR) test closest to 2L initiation. Triple positive tumors were those positive for ER, PR and HER2. Sites of distant metastasis (bone, lung, liver, brain, distant lymph nodes, contralateral breast, skin, and ovarian) were identified from mBC diagnosis and up to 30 days after 2L initiation. Multiple metastases to the same organ site were counted once; patients who had metastases in multiple sites contributed to multiple categories. The category “other metastasis” includes any organ site not explicitly listed and may include more than one organ site. Comorbidity burden was assessed according to the Charlson Comorbidity Index, a weighted sum of the presence of comorbidities over a 12 month period prior to the mBC diagnosis [28, 29].

Treatment-related variables included drug names, drug start and end dates, lines of treatment (LoTs) and reasons for discontinuation. Lines of treatment for mBC were algorithmically determined based on antineoplastic systemic agents that were abstracted from the EHR by CTRs, and categorized into drug class and subclass. In addition to start and end dates for each agent, CTRs abstracted reasons for discontinuation. All distinct reasons for discontinuing 2L therapies were reported. Patients’ first-line regimen (1L) included all drugs that started 14 days prior to and up to 45 days after the mBC diagnosis. The start date of 1L was on the day the first non-endocrine therapy was started. Subsequent LoTs were identified after discontinuation of all agents in the LoT, a treatment gap of > 365 days or after the introduction of (1) a new anti-HER2 targeted therapy; (2) a new subclass of chemotherapy, immunotherapy, or non-HER2-targeted therapy; (3) a drug in the same subclass of immunotherapy or non-HER2 targeted therapy if the reason for discontinuation of the first drug was (a) progression or (b) intolerance or toxicity without evidence of progression. Endocrine therapy introduction or discontinuation alone did not determine the beginning or end of a LoT. LoTs were generated until the end of the systemic therapy record.

Regimens containing HER2-targeted therapy (i.e. trastuzumab or a trastuzumab biosimilar, pertuzumab, T-DM1, T-DXd, lapatinib, margetuximab, neratinib, tucatinib or pyrotinib); chemotherapy (not including HER2-targeted therapy); or other targeted therapy (not including HER2-targeted therapy or chemotherapy) were further categorized as monotherapy if given alone or with endocrine therapy or as combination therapy. ADCs (T-DM1 and T-DXd) may be given as monotherapy or in combination with other antineoplastic therapies. Among patients with recurrent metastatic disease, neoadjuvant treatment was defined as systemic therapy given prior to the definitive surgery; treatment given after surgery (if applicable) and before metastatic disease diagnosis were considered adjuvant therapy.

### Outcomes

Clinical outcomes associated with 2L included time to treatment discontinuation (TTD), time to next treatment (TTNT), and real-world progression free survival (rwPFS). TTD was defined as time from index to earlier of treatment discontinuation or death; patients without a treatment discontinuation were censored at the earliest of either the date of last contact with a healthcare professional recorded in the EHR or data cut-off on February 28, 2022. TTNT was defined as time from index to the earlier of third-line (3L) initiation or death; patients who had not initiated a 3L therapy were censored at the first of last contact or data cut-off. rwPFS was defined as the time from index to the earlier of clinician-confirmed progression (based on evidence from a pathology or radiology report) or death; patients who did not experience clinician-confirmed progression or death were censored at 3L initiation, last contact, or data cut-off, whichever occurred first. The date of data cut-off was selected to allow for a potential follow-up of 12 or more months for all included patients. Date of death was determined by a validated mortality score captured via (1) hospital-based cancer registries; (2) the Social Security Death index; (3) online obituary data; (4) manually abstracted data from physician notes; and (5) health systems’ EHR [30].

### Statistical analysis

Patient characteristics, treatment patterns and outcomes were evaluated for the full study population as well as for the subset of patients who received a trastuzumab-based 2L regimen (T-based 2L), not including T-DM1. The distributions of continuous variables were summarized as medians and interquartile ranges (IQR: 25th percentile, 75th percentile), and categorical variables as frequencies and percentages. Missing and unknown values were included in the calculation of percentages. The distribution of time-to-event outcomes (TTD, TTNT, rwPFS) were estimated using the Kaplan–Meier Product Limit estimator. The number of events and censored patients was tabulated and summarized. Median time-to-event and 95% confidence interval (CI) of the median were reported in months. All data analyses were performed in RStudio [31]. The study received Institutional Review Board exemption on 2/11/2022.

## Results

**Fig. 1:**
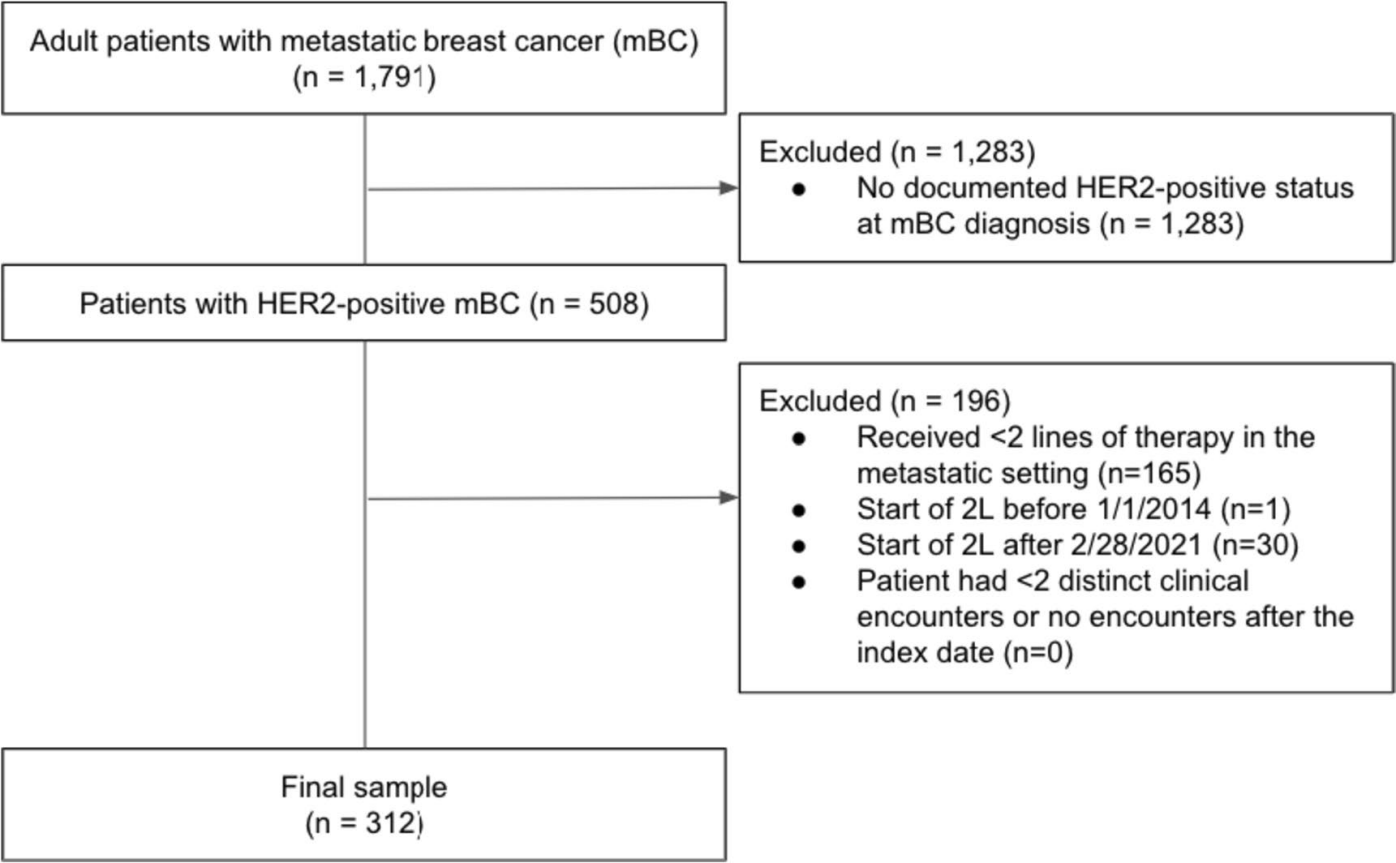
CONSORT diagram for the study cohort detailing criteria applied to reach the final sample of patients with 2 or more lines of therapy in the metastatic setting

Among 1791 mBC patients with abstracted data, 312 had HER2 + tumors and received 2 or more lines of therapy in the metastatic setting (Fig. 1). Median follow-up for all patients was 22 months (IQR, 13–37) from the index date. Median age at mBC diagnosis was 57 (IQR, 49, 64) years. The majority of patients were white (69%), non-Hispanic (86%), resided in the Midwest (89%), and were diagnosed with de novo metastatic disease (62%) (Table 1). At the start of 2L, 54% were hormone-receptor positive and 34% of patients were “triple positive” for ER, PR, and HER2. Over half of patients (56%) were postmenopausal at initial diagnosis. The Black or African American population (21%) had a median age of 53 years [IQR, 45–63] at mBC diagnosis (*median age* White: 57 years [IQR, 49–64]) and 55 years [IQR, 46–55] at 2L initiation (*median age* White: 59 years [IQR, 51–66]); more than two-thirds (69%) were diagnosed with metastatic disease at initial diagnosis (White: 62%). Patients in the T-based 2L subcohort (n = 116) had similar characteristics and are included in Table 1 with a median follow-up of 16 months (IQR, 16–45).

**Table 1:** Demographic and clinical characteristics of included patients

	All (n = 312)	T-based 2L (n = 116)
Median age (years) at index, (IQR)	59 (50, 66)	60 (50, 67)
Female, n (%)	308 (99)	116 (100)
Race, n (%)		
White	216 (69)	83 (72)
Black or African American	64 (21)	24 (21)
Other	2 (2)	2 (2)
Unknown	24 (8)	7 (6)
Non-Hispanic/Non-Latino,^a^ n (%)	268 (86)	102 (88)
Region of residence, n (%)		
Midwest	277 (89)	108 (93)
South/East	35 (11)	8 (7)
Postmenopausal, n (%)	176 (56)	63 (54)
Metastatic diagnosis,^a^ n (%)		
De novo	193 (62)	72 (62)
Recurrent	114 (37)	42 (36)
Site(s) of distant metastasis at index,^b^ n (%)		
Bone	180 (58)	62 (53)
Distant lymph node	131 (42)	46 (40)
Lung	120 (38)	41 (35)
Liver	120 (38)	39 (34)
Brain	83 (27)	30 (26)
Other	57 (18)	17 (15)
Hormone receptor positive,^a^ n (%)	169 (54)	65 (56)
Triple positive,^a^ n (%)	105 (34)	38 (33)
Charlson Comorbidity Index at mBC diagnosis,^c^ n (%)		
0	53 (17)	23 (20)
1	7 (2)	2 (2)
2 +	156 (50)	51 (44)
Unknown	96 (31)	40 (34)

^a^The denominator includes the “Unknown” category. ^b^Site(s) of distant metastasis assessed from mBC diagnosis up to 30 days after the index date. Multiple metastases at the same organ site were counted once. “Site(s) of distant metastasis” includes all that apply. The “Other” category may capture more than one site. ^c^ Calculated based on conditions present in the year prior to mBC diagnosis

### Treatment characteristics

#### Pre-metastasis treatment

Of 312 patients, 114 were diagnosed with recurrent metastatic disease (Supplementary Table S1). Nearly all (107/114, 94%) received surgery for removal of primary disease; 75% were mastectomies. Half received neoadjuvant treatment of any kind (59/114), of which 73% (43/59) received a HER2-targeted therapy. In the adjuvant setting, 75% (85/114) received any treatment; and 51% of those receiving treatment (43/85) received a HER2-targeted agent. Approximately two-thirds of all 312 patients (63%) received radiation to the primary site. Similar proportions were observed in the T-based 2L subcohort. However, within the T-based 2L subcohort only 60% (12/20) of patients with neoadjuvant treatment and 38% (13/34) of patients with adjuvant treatment received HER2-targeted therapies.

#### Systemic treatment in the metastatic setting

More than half of the 312 patients (54%) initiated 2L therapy from 2018 and onwards; 37% received only 2 LoTs, 29% only 3 LoTs, and 35% received ≥ 4 LoTs. A similar distribution was observed in the T-based 2L subcohort.

**Fig. 2:**
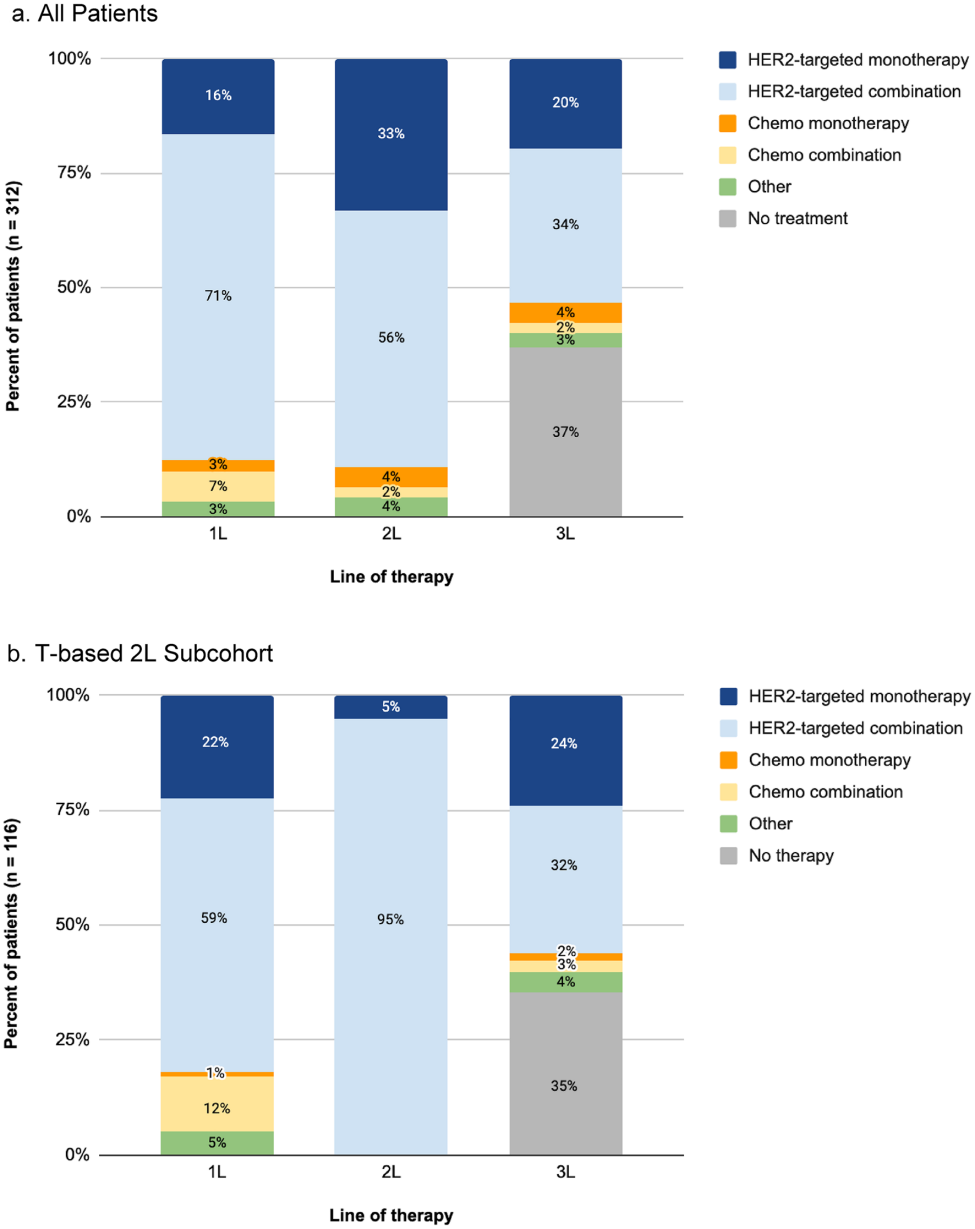
Distribution of First (1L), Second (2L), and Third-Line (3L) Regimen among all patients who received at least 2 lines of therapy in the metastatic setting (**a**) and patients who received a trastuzumab-based 2L regimen (T-based subcohort) (**b**). HER2-targeted regimen(s) include trastuzumab or a trastuzumab biosimilar, pertuzumab, lapatinib, margetuximab, neratinib, tucatinib or pyrotinib and the antibody drug conjugates (ADC) T-DM1 and T-DXd; chemotherapy-based regimens do not include HER2-targeted therapy; and other targeted therapy-based regimens do not include HER2-targeted therapy or chemotherapy. ADCs may be given as monotherapy or in combination with other antineoplastic therapies. “No treatment” includes patients who were on a 2L regimen at the end of follow-up and patients with no further documented treatment for any reason, including death and loss to follow-up

In 1L, 87% of all patients and 82% of the T-based 2L subcohort received a HER2-targeted therapy, either as monotherapy or in combination with other agents (Fig. 2). Most patients received a T-based regimen (All patients: 78%; T-based 2L subcohort: 68%). Among all patients, the three most frequent regimens in 1L were trastuzumab + pertuzumab + taxane (40%); trastuzumab + pertuzumab + taxane + carboplatin (11%); and trastuzumab monotherapy (10%). Half of patients (52%) received 1L standard-of-care treatment consisting of taxane + trastuzumab + pertuzumab (THP) with or without other agents. The most common regimens in 1L for the T-based 2L subcohort were trastuzumab + pertuzumab + taxane (28%); trastuzumab (14%); T-DM1 (9%). In 1L, 7% patients received T-DM1; 15/51 in the HER2-targeted monotherapy group and 7/222 in the HER2-targeted combination group.

Among all 312 patients, 89% received HER2-targeted therapy in 2L, of which 42% received a T-DM1-containing regimen and 37% a T-based regimen (Fig. 2). The most common regimens were T-DM1 with or without endocrine therapy (29%), trastuzumab + pertuzumab + taxane (10%), and T-DM1 + trastuzumab (8%). In 2L, 20% (64/312) received standard-of-care T-DM1 monotherapy; however, an additional 22% (68/312) of patients received T-DM1 concurrent with another therapy. In the T-based 2L subcohort, the most common regimens were trastuzumab + pertuzumab + taxane (28%); trastuzumab + pertuzumab (11%); and trastuzumab + lapatinib (6%) in 2L. ADC-based regimens were given to 43% of patients in 2L; 65/103 in the HER2-targeted monotherapy group and 65/175 in the HER2-targeted combination group.

Subsequently, 63% [197/312] of patients received 3L and most continued to receive HER2-targeted regimens (all patients, 84% [166/197]; T-based subcohort, 86% [65/75]) (Fig. 2). The majority of patients received a T-based regimen (41%; 80/197) or a T-DM1-containing regimen (23%; 46/197) in 3L. In the T-based subcohort, patients were more likely to receive a T-DM1-based regimen (44%; 33/75) or a T-based regimen (39%; 29/75) in 3L. The most common regimens in 3L were T-DM1 (19%), T-DXd (10%), and capecitabine + lapatinib (8%) among all patients and T-DM1 (35%), T-DM1 + trastuzumab (7%), palbociclib (4%) in the T-based subcohort. Among patients who received HER2-targeted monotherapy, 45/61 received an ADC, while 23/105 patients who received a HER2-targeted combination regimen did.

#### 2L reasons for discontinuation

Only 12% of patients from the full cohort and 18% in the T-based 2L subcohort remained on 2L at the end of follow-up. The majority of patients discontinued 2L (All patients, 88%; T-based 2L subcohort, 82%). Of the 274 patients who discontinued 2L in the full group, 20% died either while on 2L or after completing 2L treatment; 21 (8%) did not receive further treatment; and 72% started a new line of therapy. Similar patterns were observed in the T-based 2L subcohort (Table 2). In the full group, the three most common reasons for discontinuing 2L treatment were progression/worsening of cancer (47%); intolerance or toxicity with no evidence of progression (17%); and end of planned therapy (9%). The T-based 2L group discontinued 2L for the same top three reasons; however, the proportion of patients who completed planned therapy was 20% (Table 2).

**Table 2:** Second-line (2L) treatment attrition and reasons for discontinuation

	All (n = 312)	T-based 2L (n = 116)
Ongoing 2L at end of follow-up, n (%)	38 (12)	21 (18)
Discontinued 2L, n (%)	274 (88)	95 (82)
Initiated 3L^a^	197 (72)	75 (79)
Ongoing 2L at the time of death^a^	7 (2)	1(1)
Died after 2L, prior to 3L start^a^	49 (18)	11 (12)
No further treatment during follow-up^a^	21 (8)	8 (8)
Most common reasons for discontinuation, n (%)		
Progression/worsening of cancer^a^	128 (47)	39 (41)
Intolerance/toxicity in absence of progression^a^	47 (17)	22 (23)
End of planned therapy^a^	25 (9)	19 (20)

^a^Distribution is based on patients who discontinued 2L therapy

### 2L-associated clinical outcomes

**Fig. 3:**
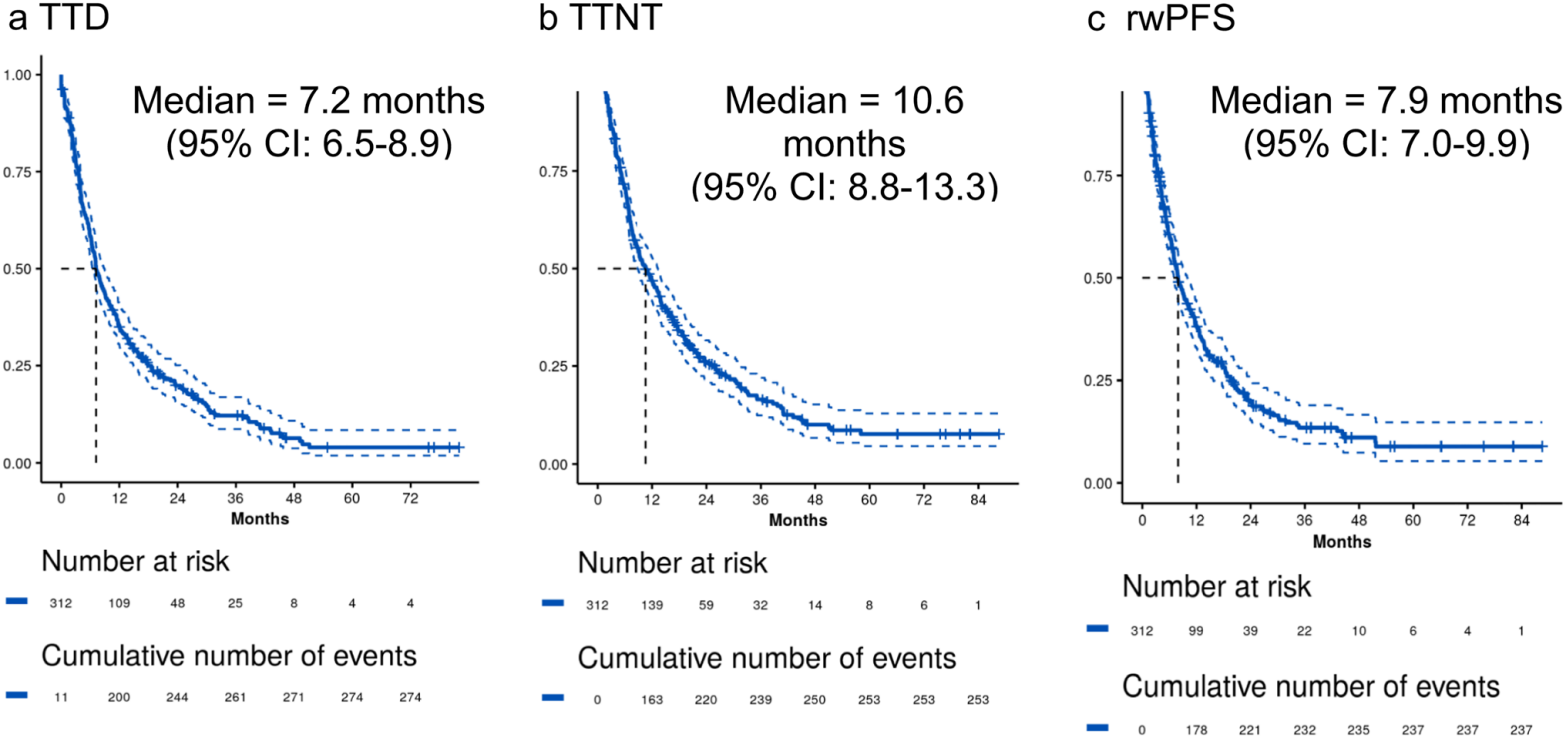
Clinical Outcomes for Second-Line Treatments in HER2 + mBC and median number of months-to-event from 2L initiation (index). **a** Time to treatment discontinuation (TTD) is the time from index to the first of treatment discontinuation or death; patients without the event were censored at the date of last contact or data cut-off (DCO), whichever came first. **b** Time to next treatment (TTNT) is the time from index to the first of 3L initiation or death; patients were censored the date of last contact or DCO, whichever came first. **c** real-world Progression-free survival (rwPFS) is the time from index to the first of clinician-confirmed progression or death; patients were censored at 3L initiation, last contact or DCO, whichever came first

Figure 3 summarizes clinical outcomes for all patients from the date of 2L initiation. The median TTD was 7.2 months (95% CI, 6.5–8.9). Median TTNT was 10.6 months (95% CI, 8.8–13.3); and median rwPFS was 7.9 months (95% CI, 7.0–9.9). Among 116 patients who received a T-based regimen in 2L, the median TTD was 10.6 months (95% CI, 7.4–14.0); median TTNT was 14.9 months (95% CI, 9.9, 22.0); and the median rwPFS was 13.6 months (95% CI, 8.3, 20.2).

## Discussion

This observational study of HER2 + mBC patients routinely treated in clinical practice across the US shows that majority of patients (88%) discontinued their 2L with a median time to treatment discontinuation of 7.2 months (95% CI: 6.5–8.9 months). Disease progression and intolerance/toxicity to current treatments were amongst the most common reasons noted for treatment discontinuation suggesting 2L effectiveness and tolerability continues to remain a challenge. Also key to note was that of the 274 patients who discontinued 2L therapy 20% died during or just after 2L therapy. Additionally, two-thirds of the 2L patients also went on to receive a subsequent therapy. The results of this study highlight the patient attrition seen in the metastatic setting and further emphasize the need for effective treatments being used at the earliest opportunity in the metastatic setting.

Our ability to compare results to other contemporary real-world studies of HER2 + mBC patients with ≥ 2 LoTs is limited to specific elements of treatment sequencing and types of outcomes. Our study found that 63% of patients who initiated 2L went on to receive subsequent therapy, which was similar to the 58% reported in a cross-sectional European chart-review study of patients in Spain, Italy, the Netherlands and the UK conducted between January and April 2016 [24]. An Italian real-world study of 1,328 patients with advanced HER2 + disease diagnosed between 2000–2020 reported a similar proportion of patients progressing to 3L (62%) [27]. In the current patient population, T-DM1 use alone or in combination with other agents in 2L was 42%, a proportion within the range of 36% to 72% of other real-world data studies [24, 25]. Differences in study populations and study period are likely to contribute to the wide range of estimates. The majority of patients (89%) received a HER2-targeted 2L regimen, consistent with 82% reported by another study of patients who initiated 2L between 2013 and 2019 in the US community setting [26]. The same study reported a median duration of 2L of 6 months, compared to our TTD of 7 months [26]. Other studies focused on a particular subgroup of patients (e.g. patients with brain metastases), limiting direct comparability of results [23]. In agreement with SEER data, this study found that Black or African American patients were more likely to be diagnosed with more advanced disease at a younger age than white patients [42], emphasizing the need to address health disparities.

The results from this study shows that in real-world practice there was a moderate adherence to standard-of-care recommendations contemporary to this study; 52% of patients received THP in 1L and 21% received T-DM1 monotherapy in 2L. It should be noted that this analysis predates the updated guidelines recommending the use of T-DXd in mBC HER2 + patients who have received a prior anti-HER2-based regimen and that the treatment patterns seen in the overall cohort and the T-based cohorts could be a reflection of the temporal changes in the treatment landscape. Beyond 1L, trastuzumab-based regimens are a common treatment choice in real-world practice even after the introduction of newer therapies (e.g. T-DM1) indicated for patients who received prior HER2-targeted treatment as seen in this study and others [26]. The continued use of trastuzumab-based regimens beyond progression while on trastuzumab may be due to evidence showing some clinical benefit with continuation compared to chemotherapy alone [32, 33]. It may also reflect the practice of conserving newer therapies for patients with poorer prognosis or only when trastuzumab-based regimens are no longer an option due to intolerance, toxicity or disease progression [34, 35]. A real-world study of patients who were treated with T-DM1 between 2013 and 2018 found that 40% of patients had metastasis at three or more organ sites and 62% had 2 or more prior treatments before T-DM1 [36]. Heterogeneity in treatment practices may also be driven by the need to balance effectiveness and toxicity concerns due to the limited evidence on treatment sequencing or the optimal number of lines of treatment in the metastatic setting [37].

For the overall study population, rwPFS of 7.9 months was within the range of results of 3 to 10 months reported in clinical trials conducted among previously treated patients in the metastatic setting [15, 17, 19, 38–40]. Patients who received a T-based 2L had a rwPFS of 13.6 months, which was longer than the median PFS of patients randomized to the trastuzumab plus chemotherapy arm of SOPHIA (4.9 months [95% CI, 4.2–5.6]) [38]. This may be due to differences in the trial’s inclusion criteria (e.g. 2 or more prior HER2-targeted therapies, 1 to 3 lines of therapies) and regimen differences. The inclusion of pertuzumab in some T-based 2L regimens may have increased median rwPFS. CLEOPATRA demonstrated a 6 month improvement in 1L PFS with the addition of pertuzumab to trastuzumab + docetaxel, emphasizing the importance of concomitant therapies in T-based regimens [41].

The strength of this study is the inclusion of contemporary data on patients treated in a more recent time period than previous studies and is unselected for specific prior treatments. Data include the longitudinal capture of all care; detailed systemic therapy information, including reasons for treatment discontinuation; and clinician-assessed progression confirmed by imaging or biopsy. The study’s limitations include those inherent in real-world data from routine care such as missing data that may result in measurement error or misclassification. To augment comprehensiveness and accuracy, the database integrates data from multiple sources additionally curated by CTRs. Patients diagnosed in the last five years are prioritized for manual curation, capturing contemporary treatment practices and outcomes to inform current areas of unmet need. Off-label combinations (e.g. T-DM1/trastuzumab) were observed, therefore the results should be interpreted with caution. The cohort may not be representative of all patients cared for in the US community health setting. The study has a relatively small sample size, mostly from the Midwest, with overrepresentation of de novo metastatic patients [43]. Additionally, patients who were prioritized for further data abstraction were diagnosed within 5 years of current date and belong to a health system within the Syapse LHN and may not be representative of all mBC HER2 + patients. Patients with de novo metastatic disease have different disease trajectories and better prognosis compared to patients who recur, which may lead to more favorable estimates of clinical outcomes [4, 44]. Future studies should further investigate treatment sequencing and outcomes in subgroups defined by factors of clinical relevance such as hormone receptor status, metastatic status (de novo or recurrent), and treatment history prior to metastatic disease diagnosis.

## Conclusions

Treatment patterns and clinical outcomes in real world US data highlights the need to provide timely 2L treatment with the most efficacious drug to allow patients to achieve longest duration on treatment and delay progression.

### Supplementary Information

Below is the link to the electronic supplementary material.

Supplementary file1 (DOCX 14 KB)

## Data Availability

We will adhere to the ethical obligations for responsible sharing of data. The data that support the findings of this study are not publicly available due to patient privacy and legal restrictions. Requests for access to the de-identified data can be submitted to research@syapse.com for review and approval.

## Abbreviations

1L: First line of therapy
2L: Second line of therapy
3L: Third line of therapy
ADC: Antibody drug conjugate
CTR: Certified tumor registrar
DCO: Data cut-off
HER: Electronic health record
IQR: Interquartile range
LoT: Line of therapy
mBC: Metastatic breast cancer
rwPFS: Real-world progression-free survival
T-based: Trastuzumab-based
T-DM1: Ado-trastuzumab emtansine
T-DXd: Trastuzumab deruxtecan
TTD: Time to treatment discontinuation
TTNT: Time to next treatment
