# Supplementary material for: A real-world study of treatment sequences and second-line clinical outcomes in patients with HER2-positive metastatic breast cancer in US community practice
Source: Int J Clin Oncol. 2024 Mar 25;29(6):780–9. doi: 10.1007/s10147-024-02492-5 (PMC11130013; doi:10.1007/s10147-024-02492-5)
Supplement: Supplementary file 1 — Supplementary file1 (DOCX 14 KB) [file 10147_2024_2492_MOESM1_ESM.docx]

**Supplementary Table 1** Pre-metastasis treatment for patients with recurrent metastatic disease

|  | **All**  (n=114) | **T-based 2L^a^**  (n=42) |
| --- | --- | --- |
| **Surgery to the primary site**, n (%) | 107 (94) | 41 (98) |
| **Surgery type^b^**, n (%) |  |  |
| Mastectomy | 74 (65) | 30 (71) |
| Breast conserving surgery | 31 (27) | 11 (26) |
| Surgery, NOS | 2 (2) | 0 (0) |
| None | 7 (6) | 1 (2) |
| **Curative radiation therapy to the primary site,** n (%) | 72 (63) | 23 (55) |
| **Site of curative radiation**, n (%) |  |  |
| Breast | 19 (17) | 7 (17) |
| Chest wall | 7 (6) | 3 (7) |
| Breast, chest wall, lymph nodes | 5 (4) | 2 (5) |
| Unknown | 41 (36) | 11 (26) |
| None | 42 (37) | 19 (45) |
| **Neoadjuvant therapy**, n (%) | 59 (52) | 20 (48) |
| *HER2-targeted neoadjuvant therapy ^c^* | *43 (73)* | *12 (60)* |
| **Adjuvant therapy**, n (%) | 85 (75) | 34 (81) |
| *HER2-targeted adjuvant therapy ^c^* | *43 (51)* | *13 (38)* |

^a^Patients in the trastuzumab-based second line of therapy (T-based 2L) group received a second-line regimen that contained trastuzumab, a trastuzumab biosimilar, or a trastuzumab combination therapy. ^b^Mastectomy includes modified radical mastectomy, total mastectomy, mastectomy, not otherwise specified (NOS), subcutaneous mastectomy; breast conserving surgery includes lumpectomy and partial mastectomy. ^c^ The proportion of patients who received a HER2-targeted therapy is based on the number of patients treated. HER2-targeted regimens are those that contain any of: trastuzumab or a trastuzumab biosimilar, pertuzumab, lapatinib, margetuximab, neratinib, tucatinib, pyrotinib or antibody drug conjugates T-DM1 or T-DXd.
